# Development and Optimization of Chitosan-Ascorbate-Based Mucoadhesive Films for Buccal Delivery of Captopril

**DOI:** 10.3390/pharmaceutics17040401

**Published:** 2025-03-22

**Authors:** Krisztián Pamlényi, Hala Rayya, Alharith A. A. Hassan, Orsolya Jójárt-Laczkovich, Tamás Sovány, Klára Pintye-Hódi, Géza Regdon, Katalin Kristó

**Affiliations:** Institute of Pharmaceutical Technology and Regulatory Affairs, University of Szeged, Eötvös u. 6., H-6720 Szeged, Hungary; pamlenyi.krisztian@szte.hu (K.P.); hala.rayya95@gmail.com (H.R.); alharith.hassan@szte.hu (A.A.A.H.); jojartne.laczkovich.orsolya@szte.hu (O.J.-L.); sovany.tamas@szte.hu (T.S.); hodiklara@szte.hu (K.P.-H.); kristo.katalin@szte.hu (K.K.)

**Keywords:** buccal drug delivery system, captopril, chitosan-ascorbate, DoE, factorial design, hypertension crisis, mucoadhesive film, QbD

## Abstract

**Background:** Captopril (CAP), an angiotensin-converting enzyme inhibitor (ACEI), is widely prescribed for managing hypertension, heart failure, and related conditions. When administered orally, CAP undergoes hepatic metabolism, resulting in a bioavailability of 60–75%. However, to bypass the first-pass metabolism and other limitations of the oral route, mucoadhesive buccal films have gained attention as a promising alternative with several advantages. The aim of this work was the formulation and optimization of chitosan-ascorbate mucoadhesive films for buccal delivery of CAP for the management of a hypertension crisis (10 mg and 20 mg) by employing quality by design (QbD) principles and the design of experiment (DoE) approach. **Materials and methods**: In the present work, chitosan (CHI) was selected as a film-forming agent due to its permeability-enhancing properties, which could be further improved through salification with ascorbic acid (AA). The polymer films were prepared by the solvent casting method. **Results**: The optimized CAP-loaded formula showed appropriate in vitro mucoadhesion force (>15 N) and breaking hardness (>14 N). The different CAP-containing films had a high drug content (>95%) with homogeneous drug distribution, thus complying with the requirements of Pharmacopeia. FT-IR and RAMAN spectroscopy analyses demonstrated successful incorporation of the drug, and interaction was observed between the excipients of the films, especially in the form of hydrogen bonds. The dissolution test showed immediate release of the API with a similar release pattern from both concentrations of CAP-loaded films. **Conclusions**: The properties of the prepared films met the predetermined critical quality attribute requirements. The optimized formula of CHI 1.4%, AA 2.5%, and glycerol 0.3% appears to be a promising buccal drug delivery system for CAP.

## 1. Introduction

The buccal administration route has gained growing attention in pharmaceutical research and industry for the delivery of a variety of active pharmaceutical ingredients (APIs) [1,2]. This route offers several advantages, including rapid onset of action, direct access to systemic circulation, and bypassing the hepatic first-pass effect; therefore, less API can be applied to achieve the same effect [3,4]. Due to this fact, side effects can be reduced. In addition, the buccal route provides a non-invasive alternative for patients who experience difficulty swallowing or those who suffer from gastrointestinal disturbances [1]. Additionally, the buccal mucosa has a relatively high permeable nature, rich vascularization, and ease of access, making it an ideal site for drug delivery [5].

Buccal drug delivery systems are available in various forms, such as tablets, gels, lozenges, sprays, and patches/films, and each of them has been designed to address specific therapeutic requirements [2,6,7]. Among these, buccal mucoadhesive films might be preferred over others by patients due to their small size, reduced thickness, high flexibility, and ease of application [3,8]. These thin films adhere to the mucin of buccal mucosa, ensuring localized or systemic drug delivery while minimizing the risk of accidental swallowing or displacement [1,9].

The preparation of buccal mucoadhesive films involves two conventional techniques, namely, solvent casting and the hot-melt extrusion method. Newer methods using the latest technologies have been investigated, such as 2D and 3D printing, electrospraying, and electrospinning [6,10,11]. However, the solvent casting method is the most commonly used manufacturing process due to the ease of the process and low cost [12]. This method comprises a number of steps, with drying of the solution being the most time-consuming step, especially in the case of aqueous solutions. However, it was reported that using a ventilated oven under certain optimized conditions reduced the drying time from around 72 h to 8 h [13].

For the preparation of mucoadhesive films, the selection of a film-forming polymer is critical to achieving the desired film properties. Among these polymers, chitosan (CHI) has garnered particular interest due to its biocompatibility, biodegradability, and inherent mucoadhesive properties because it is a cationic polymer compared to the other often-used polymers, which are anionic [14,15]. Due to this property, CHI can create a stronger bond with the anionic mucin of the buccal mucosa. CHI is a linear polysaccharide with randomly linked β-(1–4) D-glucosamine and N-acetyl-D-glucosamine. It possesses permeability-enhancing properties, which can be further improved by polymer salification with citric acid [14,16,17,18].

Due to its chemical nature, CHI dissolves under acidic conditions. Ascorbic acid (AA), also known as vitamin C, is one of the acids that can be used for the acidification of the solution [19]. AA possesses acidic hydroxyl groups, while CHI contains amino groups that can be protonated in acidic conditions, enabling the formation of the chitosan-ascorbate complex through ionic interactions. AA is essential in the human diet, is necessary for creating connective tissues and bones, and its antioxidant effect is also well known [20,21].

In the literature, several studies have investigated the applicability of chitosan in buccal drug delivery systems [19,22,23,24]. However, there are limited studies investigating chitosan-ascorbate mucoadhesive films [25,26]. One finding demonstrated the applicability of AA in CHI films, and the optimized film composition was selected based on the “One-Factor-at-a-Time” approach, the traditional method in pharmaceutical experimentation [25].

Nowadays, quality by design (QbD) principles have increasingly been used in a variety of research and industrial activities, with more importance in pharmaceutical product development [27,28]. Design of experiment (DoE), the main component of pharmaceutical QbD, is a structured and organized method for determining the relationships between input factors affecting one or more output responses through the establishment of mathematical models, and its framework consists of three successive steps: factors range finding, screening, and optimization [27,29]. In our current work, the DoE approach was applied to facilitate the formulation and optimization of chitosan-ascorbate buccal films.

In this study, captopril (CAP) was used as a model drug, as it is an angiotensin-converting enzyme inhibitor (ACEI) [30]. CAP is a member of class I in the biopharmaceutical classification system (BCS), but other authors reported that it falls into BCS class III [30]. CAP is widely used in the management of hypertension, heart failure, left ventricular dysfunction post-myocardial infarction, and diabetic nephropathy [30]. It is marketed in a tablet dosage form with variable dose strengths, namely, 12.5, 25, and 50 mg [31,32]. After oral administration, it undergoes hepatic first-pass metabolism, resulting in a bioavailability of 60–75%, which is reduced by 25 to 55% when given with food [30]. Thus, delivering CAP via buccal films can enhance bioavailability and overcome the limitations associated with the oral route of administration.

The aim of our current work is to formulate and optimize chitosan-ascorbate mucoadhesive films by applying QbD principles and using the DoE approach for buccal delivery. Depending on the preformulation results, we tried to incorporate CAP into the optimal polymer system. Furthermore, we planned to perform physical and chemical tests of these films. The final aim was to find the optimal composition that can be used as a potential buccal drug delivery system.

## 2. Materials and Methods

### 2.1. Materials

Medium- (average MW: 1,250,000 Da; batch number: 889EMW) and low- (average MW: 250,000 Da; batch number: 949JMZ) molecular-weight CHI (≥90% degree of deacetylation) were used as film-forming polymers and were purchased from Glentham Life Sciences Ltd. (Corsham, UK). Glycerol (GLY) (Ph. Eur.9.0.; ≥99.5%) was supplied by Merck (Darmstadt, Germany). AA (Ph.Eur.9.0; Lot No. D19408696) and propylene glycol (PG) (Ph.Eur.8.0; Lot No. CB1193268) were purchased from Molar Chemicals Ltd. (Halásztelek, Hungary). CAP (Ph.Eur.9.0; Lot No. 460500723) was obtained as a gift from EGIS Pharmaceuticals PLC (Budapest, Hungary). Mucin Type II from the porcine stomach was purchased from Sigma-Aldrich (Shanghai, China). Distilled water was used as a solvent in the preparation of the solutions.

### 2.2. Design of Experiments (DoE)

#### 2.2.1. Primary Screening

The statistical analysis was performed using the Statistica^®^ program, version 13 (TIBCO Software Inc., Palo Alto, CA, USA). The critical quality attributes (CQAs) selected for the study included breaking hardness (BH), elongation (EL), and mucoadhesive force (MF). The critical process parameters (CPPs) and critical material attributes (CMAs) were the amount of the solution/plate, the grade and concentration of CHI, the type and concentration of plasticizer, and AA concentration. A screening study was carried out to identify the most significant factors affecting the CQAs. Factors and their levels were selected based on the literature review, previous experience of our research group, and some primary experiments. The screening was performed using six factors at a two-level fractionate factorial design with eight runs, as shown in Table 1.

#### 2.2.2. Optimization Step

Based on the results of the screening step, further optimization was carried out using three factors at a two-level full factorial design plus one central point, as shown in Table 2.

### 2.3. Buccal Film Preparation

**Figure 1:**
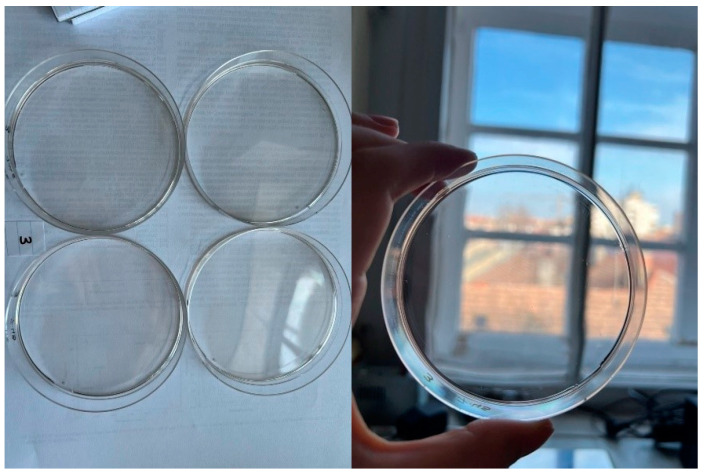
Camera photos of captopril-loaded films.

The polymeric films were prepared by the solvent casting method based on the DoE shown in Table 1 and Table 2. In the first step, AA was dissolved in distilled water, and then CHI was dissolved in the AA solution (pH = 2.51). It was mixed by an overhead VELP (VELP Scientifica, Usmate, Italy) stirrer with a propeller-type mixing rod for four hours until a clear solution was obtained. After that, CAP was added and mixed with the polymer solution. As the last step, the plasticizer (GLY or PG) (based on the dry weight of the polymer) was added to the solution and stirred for 20 min. The polymer solution was poured onto plastic Petri dishes (diameter: 9 cm, area: 63.59 cm^2^) and allowed to dry in a ventilated oven (Memmert GmbH + Co. KG, Buechenbach, Germany) under controlled conditions (temperature: 30 °C, airflow: 30% fan capacity, time: between 12 and 24 h depending on the formulations). The dried films (Figure 1) were kept in closed containers (25 ± 1 °C, 60 ± 2% RH) for further characterization.

### 2.4. Film Weights and Thicknesses

An analytical balance (Sartorius H 51, Göttingen, Germany) was used to measure film weights (n = 8) with an accuracy of 0.0001 g. To measure the films’ thicknesses, a micrometre screw gauge with a measurement range of 0–25 mm and a resolution of 0.001 mm (Mitutoyo Co. Ltd., Kawasaki, Japan) was utilized, and the average values and standard deviations of five measurements at different points on each film were calculated.

### 2.5. Moisture Content

The moisture content of films was determined using a halogen moisture analyzer (MAC 50/NH, RADWAG, Radom, Poland). Pieces of films of 4 cm^2^ were examined. The duration of the test was 8 min. Three parallel measurements were performed using a 105 °C drying temperature and medium switch-off conditions, and the results were expressed in percentage.

### 2.6. Folding Endurance

Folding endurance was manually assessed by repeated folding at the same axis using a film with a specified surface area of 4 cm^2^. The value was determined by counting the number of folds the film withstood before tearing. The test was performed three times for each formulation of films.

### 2.7. Breaking Hardness

The BH of the films was measured using a texture analyzer developed at our institute [33]. This apparatus is equipped with various sample holders and probes tailored to different types of tests. To measure the BH, the film was positioned at the bottom of the device and fixed with a sample holder. Then, a needle-like probe (3 mm in diameter) was moved down at a constant speed (20 mm/min) towards the film. The force (range of 0–50 N) required to pierce the film was recorded and is equivalent to the BH. The results were displayed as a force–time curve on a computer connected to the equipment. The test was conducted five times for each composition. The means and standard deviations were calculated [9,34].

### 2.8. Elongation

The EL was estimated based on the previous force–time curve. The time extending from the moment the needle starts to puncture the film till the puncture is completed can be used as an indication of EL and is expressed in seconds. The test was conducted five times for each formulation, and mean values and standard deviations were calculated.

### 2.9. In Vitro Mucoadhesion Test

An in vitro mucoadhesion test was carried out by the same texture analyzer with different accessories and parameters. A rod-like probe (9 mm in diameter) was used as a sample holder, and a double-faced adhesive tape was used to fix the film on the bottom side of this probe. After that, 25 µL of a mucin solution (10% *w*/*w*) was spread on a disc (35 mm in diameter) placed at the bottom of the device. Then, the film was brought into intimate contact with the mucin solution by moving the probe down towards the disc and application of a force of 30 ± 0.1 N for 30 seconds. Then, the probe was moved upwards, and the mucoadhesive force (MF) was recorded as a peak in the force–time curve. The test was repeated five times for each formulation, and the means and standard deviations were calculated [25,35].

### 2.10. Drug Content Uniformity

Drug-containing films were prepared by adding an adequate amount of the drug so that final CAP amounts of 10 mg and 20 mg per each 4 cm^2^ of the film were obtained. To assess the uniform distribution of CAP, specimens of 4 cm^2^ were cut from different parts of the film. These specimens were dissolved in 100 mL of a phosphate buffer with pH = 6.8 and stirred for 1 h. Samples were analyzed with a UV–vis spectrophotometer (Helios Alpha, Unicam, Budapest, Hungary) at λ = 208 nm wavelength (calibration equation: y = 0.0382x, x = concentration: µg/mL, y = absorbance; R^2^ = 0.9988, range of the calibration curve: 2–30 µg/mL). The average drug content of the 3 replicate samples was taken as a final reading.

### 2.11. Dissolution Test

The dissolution test was carried out with a 6-station type 2 (paddle over the disk) USP XXIV dissolution apparatus (Erweka DT700 LH, Langen, Germany) according to the requirements of Pharmacopeia [36]. A 500 mL phosphate buffer solution was used as a dissolution medium (pH = 6.8, 37 °C), and a mixing speed of 50 rpm was used during the test. In order to produce a unidirectional drug release, the film of 4 cm^2^ was placed upon a glass slide with the help of double-faced adhesive tape, and then the slide was immersed in the dissolution flask. Aliquot samples of 5 mL were collected at a predetermined time (5, 10, 15, 20, 30, 45, 60, 90, 120, 150, 180 min). The samples were analyzed by a UV–vis spectrophotometer (Helios Alpha, Unicam, Budapest, Hungary) at 208 nm wavelength. The means and standard deviations of three parallel tests were recorded.

### 2.12. RAMAN Analysis

To investigate the interactions of the ingredients, RAMAN spectra were obtained with Thermo Fisher DXR Dispersive RAMAN (Thermo Fisher Scientific Inc., Waltham, MA, USA) equipped with a CCD camera and a diode laser operating at a wavelength of 780 nm. These measurements were carried out with a laser power of 24 mW at a slit aperture of 50 µm size on a 2 µm spot size. The discrete spectra of the individual substances were collected using 6 s exposure time, and the number of exposures was 24. A smart background was used during the whole investigation. The applied spectral range was 3200–200 cm^−1^ with cosmic ray and fluorescence corrections. 

### 2.13. FT-IR Spectroscopy

Fourier transform infrared spectroscopy (FT-IR) was used to investigate the interactions between the drug and the excipients of the prepared films using an Avatar 330 FT-IR apparatus (Thermo Fisher Scientific Inc., Waltham, MA, USA) coupled with a Zn/Se horizontal attenuated total reflectance (HATR) accessory. The samples, laid on the crystal of the equipment, were scanned for absorbance in the wavelength range of 600 cm^−1^ to 4000 cm^−1^. The spectra were obtained by applying H_2_O and CO_2_ corrections with a spectral resolution of 32 cm^-1^ from 128 scans. To evaluate the results, SpectraGryph (version 1.2.15.; Dr. Friedrich Menges Software, Entwicklung, Obersdorf, Germany) was utilized.

### 2.14. Thermal Analyses

The thermal properties of captopril-containing films were measured using TGA/DSC equipment (Mettler Toledo, Greifensee, Switzerland). A piece of film (approximately 10 mg) was weighed in a 100 μL aluminium vessel and analyzed from 25 °C to 500 °C at a heating rate of 10 °C/min under a nitrogen atmosphere of 5.0 purity with a flow rate of 50 mL/min. The results obtained from 2 measurements were evaluated using the STARe^®^ SW 16.30 software of the equipment [35].

## 3. Results

### 3.1. Screening Step

BH, EL, and MF were selected as the CQAs of the mucoadhesive buccal films. Previous work in our lab has shown that the BH values of the films should be greater than or equal to 10 N to ensure comfortable handling, and MF values should be at least 7 N to ensure adequate residence time for drug absorption [37]. For EL values, we found that films with 5.3 s of elongation cannot be folded more than 80 times, whereas films with 7.45 s of EL can be folded more than 300 times, so we set 8 s of EL as the lowest limit, indicating sufficient flexibility of the films for handling [38]. Based on the literature review, previous experience of our research group [13,23], and some primary experiments, high-risk factors and their levels were selected for screening to identify the most important ones. The results of the screening step are shown in Table 3.

To the best understanding of the relationship between the independent variables and responses, the previous data were analyzed statistically using TIBCO Statistica^®^ software (version 13), and the following equations were obtained (Equations (1)–(3)). The significant factors were signed with bold letters.

BH = 27.64 + 1.87 x_1_ + **11.9 x_2_** − **3.67 x_3_** − 0.26 x_4_ − **4.09 x_5_** + **4.10 x_6_** (1)

EL = 08.30 − 0.48 x_1_ − **1.07 x_2_** + 0.50 x_3_ − 0.36 x_4_ + 0.29 x_5_ + 0.02 x_6_ (2)

MF = 11.45 + **1.81 x_1_** − 1.50 x_2_ + **2.60 x_3_** + 0.78 x_4_ + 1.13 x_5_ + 0.31 x_6_ (3)

Equations (1)–(3) represent the full polynomial equations of the no-interaction model for BH, EL, and MF, respectively. The best-fit equations were obtained by ignoring the least important factors. For the BH, the best-fit equation was obtained by ignoring x_4_ (plasticizer type) with the statistical parameters R^2^ = 0.99701, adjusted R^2^ = 0.98953, and MS residual = 2.30625. The significant factors affecting BH were CHI concentration (x_2_), amount of solution/plate (x_6_), plasticizer concentration (x_5_), and AA concentration (x_3_), referring to the arrangement of factors in order of significance. X_2_ and x_6_ had positive effects, whereas x_5_ and x_3_ had negative effects. For the EL, the best-fit equation was obtained by ignoring x_6_ (amount of solution/plate). The only significant factor was x_2_ (CHI concentration), and it had a negative effect. The statistical parameters were R^2^ = 0.96635, adj. R^2^ = 0.88223, and MS residual = 0.255925. In the case of MF, the full equation and the best-fit equation were the same, with the following statistical parameters: R^2^ = 0.99893, adj. R^2^ = 0.99248, and MS residual = 0.1225125. AA concentration (x_3_) and CHI grade (x_1_) were significant factors, and they both had positive effects.

CHI grade (x_1_) was the significant factor in the case of MF, as the CHI medium molecular weight resulted in films with a higher MF than those with a low molecular weight. However, it had relatively low importance for both EL and BH, so a medium molecular weight grade was selected for the following experiments. Polymer concentration (x_2_) was found to be the most significant factor for both BH and EL with an inconsistent effect. Additionally, it had a relatively high importance for MF, so it was selected to be included in the following optimization step. Similarly, AA concentration (x_3_) was significant for MF and BH with a paradoxical effect, and it showed a high importance for EL. As the fourth factor (x_4_) showed low importance for the three responses, GLY was chosen as the plasticizer in the following preparations. However, its concentration (x_5_) demonstrated important effects on the film properties, and it was reported that using higher amounts of a plasticizer resulted in higher retained moisture content, which could improve the mechanical properties of the films; therefore, x_5_ was included in the optimization step for further investigation [39]. The amount of the casted solution/plate (x_6_) had positive effects on the three responses, and it significantly affected the BH; thus, its higher level was selected and used in the following preparations. To conclude, three factors were fixed (x_1_, x_4_, and x_6_), and further optimization was done for the remaining factors (x_2_, x_3_, and x_5_).

### 3.2. Optimization Step

The results of the film optimization step are shown in Table 4. Equations (4)–(6) represent the full polynomial equations of the three responses. The significant factors were signed with bold letters.

BH = **14.93** − 5.29 C + **9.50 x_2_** − 2.53 x_3_ − **5.84 x_5_** + 0.35 x_2_x_3_ − **5.35 x_2_x_5_** + 0.55 x_3_x_5_ − 0.41 x_2_x_3_x_5_ (4)

EL = **09.96** + 0.44 C − **1.69 x_2_** + 0.40 x_3_ + 0.38 x_5_ + 0.41 x_2_x_3_ − 0.11 x_2_x_5_ − 0.04 x_3_x_5_ + 0.12 x_2_x_3_x_5_ (5)

MF = **08.06** + 3.51 C − 4.42 x_2_ + 0.98 x_3_ − 0.46 x_5_ − 1.04 x_2_x_3_ − 1.93 x_2_x_5_ − 0.43 x_3_x_5_ + 0.62 x_2_x_3_x_5_ (6)

The best fit of the BH polynomial equation was obtained by ignoring the interaction factor (x_2_x_3_), and the statistical parameters were 0.99924, 0.99304, and 0.9870125 for the R^2^, Adj. R^2^, and MS residual, respectively. CHI concentration (x_2_), GLY concentration (x_5_), and their interaction factor (x_2_x_5_) significantly affected BH. As shown in Table 4, a higher concentration of CHI led to films with higher BH values, and a higher concentration of GLY led to films with lower BH values, which agreed with previous reports [40,41]. The explanation for this is that increasing the polymer concentration results in films with stronger mechanical properties, while GLY, due to its hygroscopicity, increases the moisture content of films, resulting in films with a lower BH.

The best-fit equation for EL was obtained by ignoring the interaction factor (x_3_x_5_). The values of the R^2^, Adj. R^2^, and MS residuals were 0.99952, 0.99619, and 0.0128, respectively. CHI concentration (x_2_) was the only significant factor, and a higher concentration of CHI led to films with lower values of EL, in agreement with the findings of Salehi et al. [40]. This could also be explained by the stronger mechanical properties of films due to the higher CHI concentration; consequently, the film was less able to stretch, which caused the lower EL.

In the case of MF, the full polynomial equation was the best-fit equation, and there was no significant factor affecting this response. The values of the R^2^, Adj. R^2^, and MS residuals were 0.99319, 0.94548, and 1.49645, respectively. As the models’ curvatures was not significant, the design and the three-way models were adequate for explaining the behaviour and effects of these factors on the responses within the experimental domain, without the need for more advanced or complex designs.

### 3.3. Design Space of the Optimized Film Formulation

**Figure 2:**
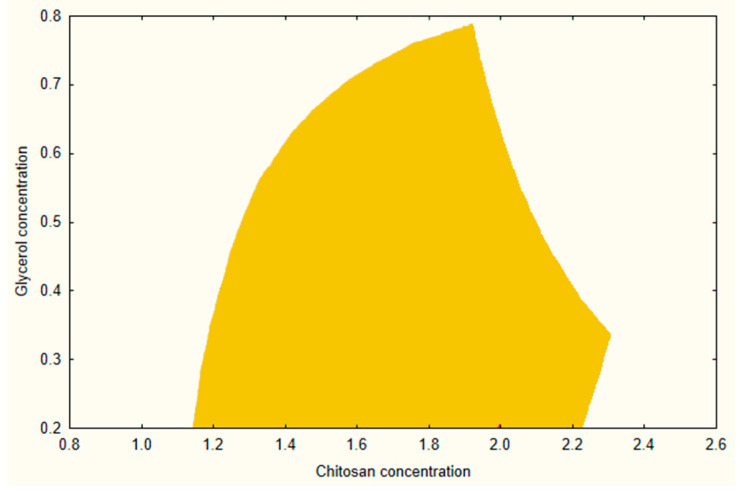
Design space of the CHI-based mucoadhesive buccal film (AA concentration = 2.5%).

A design space was created using a combination of prior experimental data and modelling while maintaining the same acceptance ranges for BH, EL, and MF as discussed during the screening process. Figure 2 illustrates the design space at the medium level of AA (2.5%). From this design, a composition of CHI 1.4%, AA 2.5%, and GLY 0.3% was selected as an optimized formula for further investigations and loading of the API.

### 3.4. Drug-Loaded Films

Following the optimization process, CAP was incorporated in amounts of 10 mg/4 cm^2^ and 20 mg/4 cm^2^ into the optimized formula, and the physical and mechanical properties were investigated. Table 5 shows the results of the physical and mechanical properties of the optimized drug-free and drug-loaded films.

The results for the drug-free and drug-loaded films fulfilled the predetermined CQAs. The incorporation of API into buccal films appeared to influence the physical and mechanical properties of the films significantly (Table 5).

The thickness of the CAP-containing films increased by increasing the CAP concentration. In general, the thicknesses of all films were within the range of the recommended buccal film thickness (between 50 and 1000 µm), with low deviations from point to point within a single film [33]. It was noticed that film weight was directly proportional to the content of the API. Moisture content values were similar for the drug-free and drug-loaded films.

It was apparent that the addition of the drug increased the flexibility of the film and featured as a significant increase in EL and a significant reduction in BH. This reduction in the BH contrasted with the findings of Hassan et al., which could be attributed to the different APIs they used, which could result in different interactions between the film-forming polymer and the API [13]. Increasing CAP concentration led to an apparent reduction in the BH while the effect on the EL was limited. However, the BH values of the drug-loaded films were well above the predetermined lower limit (10 N), ensuring suitable handling [37].

In vitro MF of the films was also influenced by the presence of the API. The presence of CAP increased the in vitro mucoadhesivity of the films significantly. This phenomenon can be explained by the interaction between the components of the films. In the film without CAP, the GLY created an interaction with the CHI, and therefore, less of the functional group remained in the system to bind to the mucin. In the case of the drug-loaded film, GLY can also interact with CAP, so in these films, more functional groups of CHI could be found, which can bind to the mucin strongly. These results correlate with what is reported in the literature [40].

It can be said that the presence of the API influenced the physical and mechanical properties of the films positively, making them more suitable for application to the buccal mucosa.

### 3.5. RAMAN Analysis

**Figure 3:**
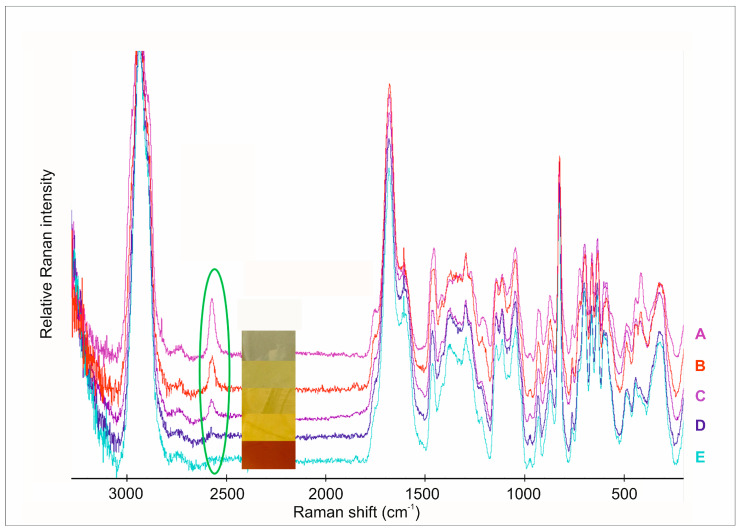
RAMAN spectra of buccal films (A: 1.25 mg CAP content; B: 2.5 mg CAP content; C: 5 mg CAP content; D: 10 mg CAP content; E: 20 mg CAP content). Rounding with green: the difference in the peak intensity at 2567 cm^−1^ is assigned to the thiol group in the buccal films with different CAP contents.

**Figure 4:**
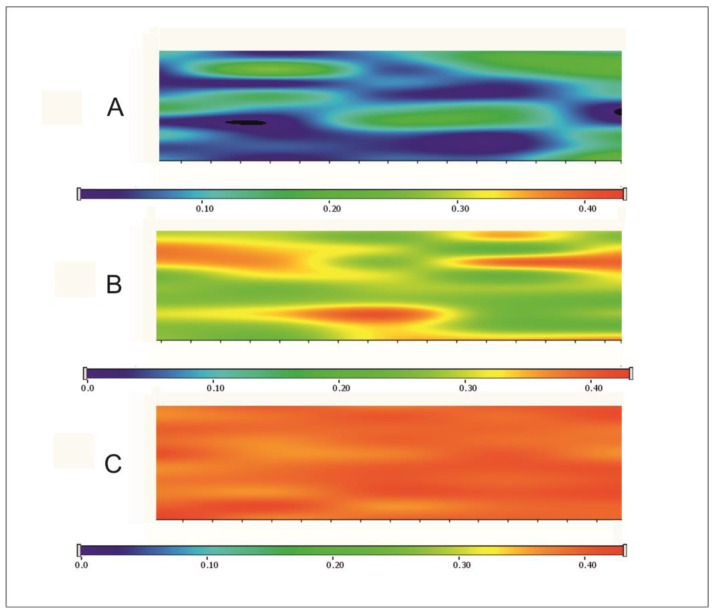
Chemical maps of buccal films with different CAP contents profiled with the peak of CAP at 2567 cm^−1^ (vibration of S-H). A: 5 mg CAP content film, B: 10 mg CAP content film, C: 20 mg CAP content film.

In this investigation, we analyzed the individual spectra of the polymer films with different amounts of the API (Figure 3). Chemical mapping was used to analyze the distribution of CAP in the films (Figure 4) [39,41]. The main aim of this analytical method was to look for interactions between the components of films. Raman spectroscopy is also suitable for the investigation of surface chemical mapping and the appearance or disappearance of special groups, so the secondary aim of this investigation was to analyze the chemical mapping of different CAP-concentrated films.

Generally, the S-H stretching vibration gives strong polarized bands in Raman spectra (Figure 3), while the band due to the S-H stretching vibration in the infrared spectra is weak and may be missed in dilute solutions [42]. It is easily recognized in the region of 2600–2540 cm^−1^ since this region is relatively free of other absorption bands. Due to the O-H stretching vibration, carboxylic acids also have bands in this region, forming a broad complex pattern. Hydrogen-bonding effects are much higher for the O-H groups than they are for the S-H group. The stretching vibrations of the S–H group at 2567 cm^−1^ were not visible in the films with 1.25 mg CAP content. As the API content increased, the intensity of S-H stretching also increased without any shifts. This means that the S-H bond appeared again in these samples. These changes were also completely followed by the colour changes in the films. In films with low CAP content, the colour was dark yellow, which indicates an oxidative interaction of the API and ascorbic acid. The increasing API content caused the colour to change and brightened the colour, which means S-H groups were in excess. The chemical maps of the films with different API content show this phenomenon.

Figure 4 shows the chemical map of films containing different amounts of the API. Chemical mapping was profiled to the peak at 2567 cm^−1^, where the thiol group can be found in the films. The blue colour suggests the presence of the S-H group of CAP at approximately 10%, while the green colour shows between 20–30% of presence at S–H stretching vibration. The red colour suggests that the presence of the S-H bonding is more than 40%. To analyze the results, it can be said that the distribution of the API in the films was completely homogenous. Moreover, in the 20 mg CAP content films, the S–H group can be found with a higher probability, which was supported by the interaction analyses. The API shows a totally homogenous distribution in this composition.

### 3.6. FT-IR Spectroscopy

**Figure 5:**
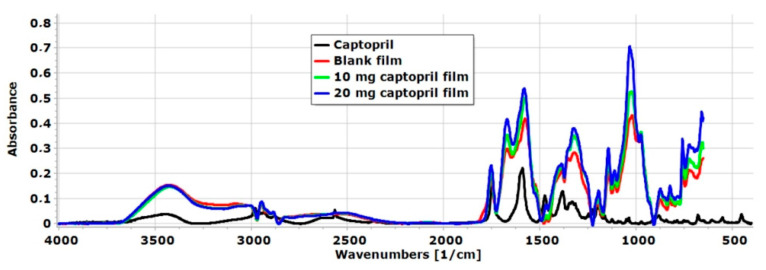
FT-IR spectra of raw materials and film formulations in the case of the blank film and CAP-containing films.

FT-IR spectra of raw API and the different chitosan-ascorbate films are presented in Figure 5. A wide spectral peak was observed in the blank and CAP-containing films at 3416 cm^−1^, which is related to the stretching vibration of OH- groups of AA, GLY, and CHI as well [25,34]. Moreover, at 3370 cm^−1^, the N-H stretching vibration of CHI appeared in the polymer film [43]. The intensity of this peak was very high, so it can be concluded that hydrogen bond interactions were formed between the N-H groups of CHI and the OH groups of GLY [44]. At 2567 cm^−1^, a sharp peak appeared in the spectra of CAP, which is related to the S-H stretching vibration [45]. This peak disappeared in the polymer film system due to interactions that were created between the other excipients of the films in the form of hydrogen bonds. At the same time, the presence of the API was demonstrated in the polymer films by RAMAN spectroscopy, so the API can be found in the chitosan-ascorbate polymer film without significant chemical changes or decomposition.

At 1477 cm^−1^, a spectral peak manifested in the spectra of CAP. At this wavenumber region, based on the literature, the asymmetric CH_3_ band is located between 1470 cm^−1^ and 1430 cm^−1^ [45]. In the chitosan-ascorbate film without API, this peak was not found, but in both API-containing films, this peak was manifested, so it can be said the API was successfully incorporated into the polymer film system. Overall, the FT-IR analysis indicates a proportional relationship between the CAP concentration and peak intensities in the FT-IR spectra. Interactions between the components of the films and the FT-IR analysis confirmed the successful incorporation of CAP into the chitosan-ascorbate films.

### 3.7. Thermal Analysis

**Figure 6:**
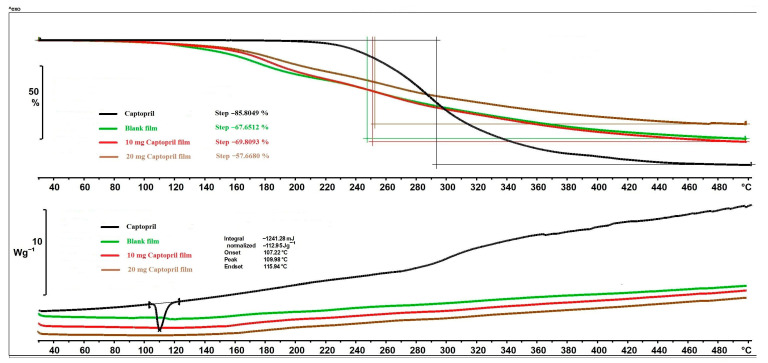
Thermal properties of API and different film compositions (TGA, DSC).

The thermal behaviour of CAP and different films can be seen in Figure 6. The mass loss of API started above 215 °C and reached 85.80% until the end of the analysis. The mass loss of polymer films occurred in one step as well, but it started at 83 °C due to the excipients of the films, especially the GLY [31]. The mass loss of blank film and 10 mg CAP film were not significantly different, but in the case of the 20 mg CAP film, the mass loss was lower. In the case of the 20 mg CAP, the chemical interaction between the polymer matrix and API may have formed to a greater extent and resulted in the lower mass loss. Concerning the DSC thermograms (Figure 6), CAP had a sharp and clearly distinguishable endothermic peak at 109.98 °C, indicating the melting point. In the CAP-containing films, the peak of CAP totally disappeared, which leads to the conclusion that the CAP was distributed in an amorphous/soluble form within the polymeric matrix. At higher temperatures, no more peaks appeared in the DSC curves of different polymer film compositions.

In summary, decomposition processes were observed above 80 °C in the polymer films, so the prepared films can be considered thermally stable up to this temperature. Furthermore, the drying temperature did not negatively affect the properties of the films, so the films could even be dried at higher temperatures.

### 3.8. Drug Content Uniformity 

The 10 mg CAP films contained 96.59% ± 5.71 (*n* = 3), and the 20 mg CAP films had 95.84% ± 4.09 (*n* = 3) of the theoretical amount of the API. These results showed uniform distribution of the drug, which agrees with and confirms the RAMAN mapping results. It can be said that these results meet the suitable extent of content uniformity according to the European Pharmacopeia (Ph.Eur.), United States Pharmacopeia (USP), and British Pharmacopoeia (BP) [46].

### 3.9. Dissolution Test

**Figure 7:**
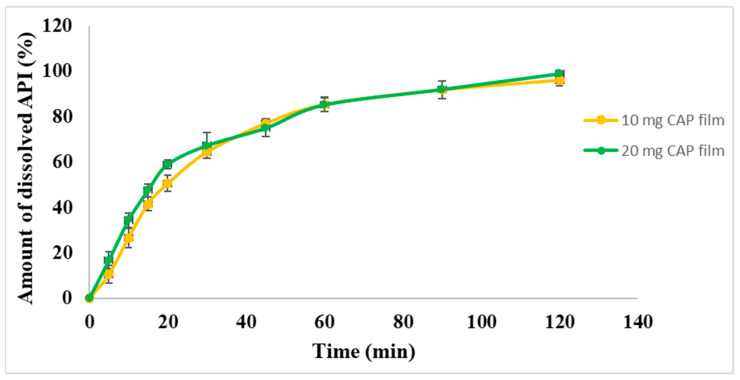
Dissolution curve of the different CAP-loaded films.

An in vitro drug release study for the drug-loaded films was performed. The cumulative % of the released drug is shown in Figure 7. More than 50% of the contained API was released in about 20 min from both types of film. After 20 min, CAP was released from both films continuously as a saturation curve according to first-order kinetic. In the indication of hypertension crises, the release of API should be fast, but in the market, the most commonly used dosage form is the per os tablets. In the case of per os tablets, the medicine should disintegrate first in the stomach, followed by dissolution and absorption of the API into the systemic circulation to normalize the blood pressure. In the case of our films, the onset of action is expected to be faster because the API is released quickly and provides a continuous release of the CAP, which can reduce blood pressure rapidly, and the effect remains for a longer period of time. It was noted that the pattern of drug release was almost the same for both concentrations of CAP-loaded films. Moreover, immediate release was achieved with both formulations (10 mg and 20 mg), which means they could be potentially applied in a buccal drug delivery system.

## 4. Conclusions

In the present work, we successfully formulated polymer films for buccal delivery using the QbD principles and the DoE approach. After the screening step, the levels of the most important factors were optimized in the form of a design space, from which an optimum composition was selected (CHI 1.4%, AA 2.5%, GLY 0.3%) to load the API. To this composition, CAP was incorporated in 10 mg and 20 mg amounts. Based on the performed tests, it can be said that the physical and mechanical properties of the films were significantly influenced by the presence of the API. CAP increased the thickness, EL, and in vitro mucoadhesivity of the films, but it decreased the BH. Overall, the thickness, BH, and in vitro MF met the predetermined requirements, so both compositions are suitable for application on the buccal mucosa. Chemical interactions were observed between the components of the films by RAMAN spectroscopy, and this finding was confirmed by FT-IR spectroscopy without signs of API decomposition. The RAMAN mapping showed homogeneous API distribution in the film, and the drug content test supported these results. Based on thermal analyses, CAP was distributed in an amorphous/molecularly dispersed form in the polymer films, and the prepared films can be considered thermally stable up to 80 °C, so the drying temperature can be increased to speed up the drying process. The in vitro drug dissolution test showed immediate and continuous drug release from the films, so the CAP-containing films can be applied in hypertension crisis cases to normalize blood pressure.

## Figures and Tables

**Table 1:** Fractionate factorial 2^(6-3)^ screening design containing six factors at two levels and eight runs; * 1: PG, 2: GLY.

Formula No.	Chitosan Grade (Average Mwt) (x_1_)	Chitosan Conc.% (*w*/*w*) (x_2_)	Ascorbic Acid Conc.% (*w*/*w*) (x_3_)	Plasticizer Type * (x_4_)	Plasticizer Conc.% (*w*/*w*) (x_5_)	Film Casting Amount (g/cm^2^) (x_6_)
F1	Low	1	2	2	0.50	0.46
F2	Medium	1	2	1	0.25	0.46
F3	Low	2	2	1	0.50	0.35
F4	Medium	2	2	2	0.25	0.35
F5	Low	1	3	2	0.25	0.35
F6	Medium	1	3	1	0.50	0.35
F7	Low	2	3	1	0.25	0.46
F8	Medium	2	3	2	0.50	0.46

**Table 2:** Three factors at two-level full factorial design with a central point for the film optimization step.

Run	x_2_: CHI (*w*/*w* %)	x_3_: AA (*w*/*w* %)	x_5_: GLY (*w*/*w*_CHI_ %)
1	1	2	25
2	2.5	2	25
3	1	3	25
4	2.5	3	25
5	1	2	75
6	2.5	2	75
7	1	3	75
8	2.5	3	75
9	1.75	2.5	50

**Table 3:** The results of the fractionate factorial screening design of BH, EL, and MF. The results are presented as mean ± SD (*n* = 5).

Formula No.	BH (N)	EL (s)	MF (N)
F1	16.58 ± 3.42	9.05 ± 0.74	10.87 ± 2.48
F2	30.44 ± 3.76	8.73 ± 0.58	10.43 ± 2.29
F3	34.13 ± 7.59	8.09 ± 1.18	5.46 ± 1.16
F4	44.09 ± 4.33	5.34 ± 0.54	8.62 ± 1.24
F5	10.65 ± 2.12	9.94 ± 0.54	12.95 ± 0.67
F6	5.30 ± 2.05	9.78 ± 0.83	17.52 ± 0.95
F7	41.75 ± 4.05	8.03 ± 1.15	9.27 ± 1.76
F8	38.20 ± 5.47	7.46 ± 0.88	16.45 ± 2.43

**Table 4:** The results of three factors at two-level full factorial design with a central point for the film optimization. The results are presented as mean ± SD (*n* = 5).

Run	BH (N)	EL (s)	MF (N)
1	9.77 ± 1.66	11.01 ± 1.63	7.94 ± 2.32
2	37.93 ± 11.37	7.29 ± 0.89	6.27 ± 2.47
3	2.07 ± 0.67	11.31 ± 2.36	14.08 ± 1.11
4	33.29 ± 7.93	8.73 ± 0.69	5.76 ± 2.13
5	6.86 ± 0.89	12.31 ± 1.19	12.99 ± 2.09
6	15.28 ± 9.47	7.65 ± 1.33	1.13 ± 1.78
7	3.03 ± 1.20	11.96 ± 1.21	14.91 ± 1.45
8	11.20 ± 4.31	9.42 ± 1.52	1.38 ± 2.08
9	25.38 ± 3.73	9.61 ± 0.97	13.92 ± 3.69

**Table 5:** Physical and mechanical properties of the optimized formula (CHI 1.4%, AA 2.5%, GLY 0.3%) for the drug-free and drug-loaded films. The results are presented as mean ± SD (*n* = 5).

	Thickness (µm)	Weight (mg)	Moisture Content (%)	Breaking Hardness (N)	Elongation (s)	Mucoadhesion Force (N)
CAP-free films	143 ± 23	77.59 ± 08.62	3.25 ± 0.59	30.36 ± 3.97	9.99 ± 0.49	8.50 ± 2.15
10 mg CAP films	157 ± 14	93.69 ± 07.56	3.97 ± 0.42	18.80 ± 3.16	11.45 ± 0.41	15.09 ± 2.37
20 mg CAP films	192 ± 22	108.0 ± 13.26	3.46 ± 0.58	14.43 ± 1.96	11.99 ± 1.03	18.51 ± 0.90

## Data Availability

Data are contained within the article.

## Abbreviations

The following abbreviations are used in this manuscript:

API	Active pharmaceutical ingredient
CAP	Captopril
CHI	Chitosan
GLY	Glycerol
AA	Ascorbic acid
PG	Propylene glycol
QbD	Quality by design
DoE	Design of experiment
FT-IR	Fourier transform infrared spectroscopy
HATR	Horizontal attenuated total reflectance
SD	Standard deviation
ACEI	Angiotensin-converting enzyme inhibitor
BH	Breaking hardness
EL	Elongation
MF	Mucoadhesive force
C	Curvature
TGA	Thermogravimetric analysis
DSC	Differential Scanning Calorimetry
CPPs	Critical process parameters
CMAs	Critical material attributes
CQAs	Critical quality attributes
Ph. Eur	European Pharmacopeia
USP	United States Pharmacopeia
BP	British Pharmacopoeia
